# Secondary prevention of silicosis and silico‐tuberculosis by periodic screening of silica dust exposed workers using serum club cell protein 16 as a proxy marker

**DOI:** 10.1002/hsr2.373

**Published:** 2021-09-22

**Authors:** Kamalesh Sarkar, Sarang Dhatrak, Bidisa Sarkar, Umesh Chandra Ojha, Pankaja Raghav, Avinash Pagdhune

**Affiliations:** ^1^ Director ICMR ‐ National Institute of Occupational Health Ahmedabad India; ^2^ Department of Poison Information Center ICMR‐National Institute of Occupational Health Ahmedabad India; ^3^ Department of Community Medicine Kalinga Institute of Medical Sciences Bhubaneswar India; ^4^ Department of Pulmonary Medicine ESIC PGIMSR & Hospital New Delhi India; ^5^ Department of Community Medicine & Family Medicine All India Institute of Medical Sciences Jodhpur India; ^6^ Department of Biochemistry Tata Memorial Centre Advanced Centre for Treatment, Research and Education in Cancer Navi Mumbai India

**Keywords:** biomarker, lung damage score, screening tool, secondary prevention, serum CC‐16

## Abstract

**Background and Objectives:**

Silicosis is a neglected and widely prevalent occupational disease in India and several other countries such as China, South Africa, Brazil, etc. It is an irreversible, incurable, and progressive disease with high morbidity and mortality, which is mostly caused by occupational exposure to silica dusts. Silicosis is usually detected at an advanced stage, when effective intervention is not possible. But early detection appears to be a cost‐effective way to control it. There is a need for some suitable biomarker, which could detect silicosis at an early stage for further necessary intervention. This study aimed to estimate the lung damage in silicotic subjects and its relationship with serum CC16 as a proxy marker. The ultimate objective was to explore whether CC16 could be used as a screening tool for early detection of silicosis.

**Methodology:**

Radiographs of 117 workers having radiological evidences of silicosis were evaluated in accordance with International Labour Organisation (ILO) Classification of chest radiographs and were categorized as mild, moderate, and severe lung damage using a lung damage scoring system, made for the purpose of this study. The concentration of CC16 in serum was determined by enzyme‐linked immunosorbent assay.

**Result:**

It was observed that serum CC16 values were significantly decreased in relation to increasing lung damage. The mean ± standard deviation (SD) serum CC16 value in mild lung damage group was 8.4 ± 0.87 ng/mL as compared to 4.0 ± 2.10 ng/mL in moderate and 0.7 ± 0.21 ng/mL in high lung damage groups. On the other hand, CC16 value of control (healthy) population was found to be 16.3 ± 3.8 ng/mL.

**Conclusion:**

Result of the study concluded that serum CC16 might be used as a periodic screening tool for early detection of silicosis and for it's secondary prevention. It may be viewed as a new approach toward control of silicosis, and an appropriate policy may be adopted.

## 1 INTRODUCTION

Respiratory public health is currently facing several challenges such as increasing burden of chronic obstructive pulmonary diseases (COPDs) due to growing air pollution, disturbing occurrences of influenza epidemics, increasing multidrug‐resistant tuberculosis (MDR‐TB) etc.^1^ On the top of it, the entire world is currently facing a highly distressing pandemic caused by severe acute respiratory syndrome coronavirus 2 (SARS‐CoV‐2). With this backdrop, it is difficult to accept that a preventable occupational disease such as silicosis continues to threaten the health of so many people worldwide just due to lack of required initiatives for it's control. Silicosis is a widely prevalent occupational lung disease caused by inhalation of crystalline silica dust and is marked by inflammation with scarring in the form of nodular lesions on the lungs.^2^ Common morbidity due to silicosis includes COPD, asthma, hypersensitivity pneumonitis, nephropathy, stomach cancer, etc.^3^, ^4^, ^5^ India has a huge burden of silicosis with an estimated 8.5 million workers in the year 1999 at risk of silicosis as per data available on national health portal.^6^ It may have at least doubled in 20 years, in view of increased population and industrialization. Many studies have been conducted among silica‐exposed workers depicting the prevalence of silicosis that ranged between 12% and 50%.^7^, ^8^, ^9^, ^10^, ^11^ Presently, silicosis is detected on the basis of opacities present over chest radiographs by using “ILO Classification of Radiographs for Pneumoconiosis,” which often makes the diagnosis delayed as it is asymptomatic or mildly symptomatic in the initial stage of the disease and often ignored by the workers. Since silicosis is an incurable, irreversible, and progressive disease with high morbidity and premature mortality, early detection appears to be the only way for secondary prevention of it. Hence, there is a need for some suitable biomarker, which can detect silicosis among dust‐exposed workers in its early stage, and thereby the workers could be identified and removed from further exposure to silica dust. In this regard, Indian Council of Medical Research–National Institute of Occupational Health (ICMR–NIOH), India, had conclusively established that serum club cell protein 16 (CC‐16) could be used as a biomarker for early detection of silicosis.^12^ However, the earlier study did not establish the relationship between various grades of lung damage and its corresponding serum CC16 value. Hence, an observational study was conducted to categorize the lung damage based on approximate quantification of silicotic damage using chest radiograph to mild, moderate, and high/severe and assessing its relationship with serum CC16 level. The ultimate objective of this study is to investigate whether serum CC16 could be used as a proxy marker/predictor and as a periodic screening tool among occupational silica dust–exposed workers for early detection of silicosis. Early indication of silicosis through periodic assessment of serum CC16 among workers with history of occupational silica dust exposure appears to be an useful method. This may be confirmed by the chest X‐ray for secondary prevention of silicosis as well as for other purposes such as notification to the local authority and compensation to be paid to the victims as per law of the country. Hence, this study was initiated.

## 2 METHODOLOGY

This was an occupational health clinic–based cross‐sectional study. The participants were workers of stone mines and stone quarries and living in the neighboring areas of their workplaces in Faridabad, Haryana and Jodhpur, Rajasthan. One hundred and forty‐nine subjects with and without radiological evidence of silicosis were subjected for this study. Of them, 32 subjects were healthy controls with no history of occupational exposure to silica dust. The radiographs of both 117 workers with evidence of silicosis and 32 healthy controls were evaluated using International Labour Organisation (ILO) radiography guidelines for detection of silicosis. Evaluation was performed for approximate quantification and categorization of lung damage.^13^ Each radiograph was examined for quality of X‐ray plate. Following this, acceptable X‐ray plates were categorized in to mild, moderate, and severe silicotic lung damage. For determination of lung damage, following four factors were considered: (a) Size of small opacities (X), (b) profusion of opacities (Y), (c) zones of lung involved (Z), and (d) presence of large opacities (L). Small opacities were determined by comparison with standard radiographs provided by ILO and recorded as one of the categories: 0, 1, 2, or 3. Shapes and sizes were determined by comparison with standard ILO radiographs. The predominant shapes and size of the regular small and round opacities were recorded as p, q, and r depending on size of the opacity. (*p =* < 1.5 mm, q = 1.5 ‐ 3.0 mm, and r  => 3.0 ‐ 10 mm). Similarly, if shapes are irregular, it is denoted as s, t, and u but with same values like p, q, and r). Large opacities are recorded as A, B, and C. (A = 10 ‐ 50 mm, B = > 50 mm, but smaller than area of right upper zone of lung and C = > area of right upper zone). The findings were noted in a standardized ILO radiograph reading sheet.

### 2.1 Approximate quantification of lung damage

For approximate quantification of lung damage, following factors were considered:

1. Size of the small opacity (X)
2. Size of large opacity (L)
3. Profusion of opacity (Y)
4. Zones of lung involved (Z)

#### 2.1.1 Size of small opacity (X)

Amount of lung damage was calculated using size of opacity on chest X‐ray. The size and shape of small opacities were scored as *p* = 1, q = 2, and r = 3. If more than one form (shape/size) of opacities was identified (mixed opacities), then the most common opacities were termed as primary opacity, while the next common opacities were secondary opacity. In case of mixed opacities, scoring was carried out as p/q = 1.5, q/r = 2.5, etc. Irregular small opacities are also categorized as s = 1, t = 2, u = 3, s/t = 1.5, etc.

#### 2.1.2 Size of large opacity (L)

Weightage of lung damage was given as per large opacity: A = 1, B = 2, and C = 3.

#### 2.1.3 Profusion of opacity (Y)

Based on the profusion of opacities, weightage is given as category 1 = 1 (low translucency on X‐ray), category 2 = 1.5 (moderate translucency on X‐ray), and category 3 = 2 (high translucency on X‐ray).

#### 2.1.4 Zones of lungs involved (Z)

Weightage is given according to number of zones involved. If one zone is involved, score given is 1 and if all six zones involved score given in 6.

### 2.2 Categorization of approximate lung damage using lung damage score (LDS)

The total lung damage score (LDS) was obtained by multiplying small opacities (X) with profusion of opacities (Y) and number of affected lung zones (Z). To this, score of large opacity (if any) is added (L).

Lung Damage Score (LDS) = (X*Y * Z) + L.

Using above scoring system, the extent of lung damage was categorized as:

Severe/high when LDS = > 15, moderate when LDS = 7 to 15, and mild when LDS = 1 to 6.

### 2.3 Blood sample collection

About 5 mL blood sample was collected from each study subjects for estimation of serum CC16 by the trained laboratory technicians from the eligible and willing study participants. Relevant demographic information was also collected from each subject using a pretested questionnaire. Study questionnaires along with blood samples were transported to the laboratory of ICMR‐NIOH for further processing. Concentration of CC16 in serum was determined by enzyme‐linked immunosorbent assay (ELISA) method.

### 2.4 Data management

Data were edited on the same day following collection and transported at ICMR—National Institute of Occupational Health. Data were entered and analyzed using Epi Info software, Windows‐7.2 version.

### 2.5 Institute ethics committee approval

The study was approved by the Institutional Ethics Committee (IEC) of ICMR‐NIOH. A written informed consent was obtained from all the study participants before initiating this study.

## 3 RESULTS

**FIGURE 1:**
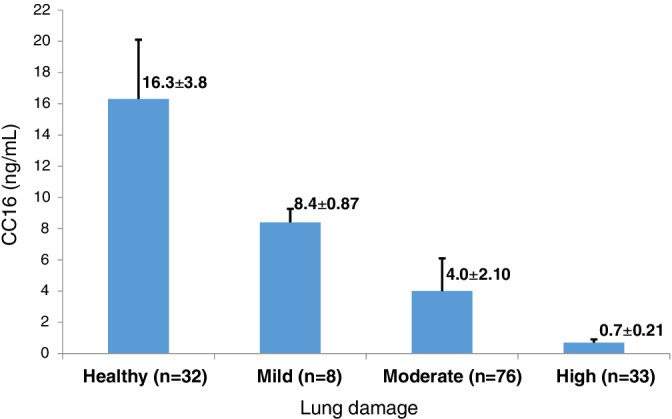
Serum CC16 values in healthy, mild, moderate, and high lung‐damaged categories

Mean age, duration of exposure, subjects with lung damage scores in various lung damage categories, and serum CC16 levels are shown in Table 1. Mean age of silicotic subjects varied between 46.2 and 50.5 year and that of healthy subjects was 37.5 year. All study subjects were males. Mean work duration for mild, moderate, and severe silicotic subjects was 28.6, 21, and 19.8 years, respectively. The result showed that out of total 117 silicotic participants, mild lung damage was observed in eight workers (6.8%), whereas moderate and high/severe lung damage were observed in 76 workers (65%) and 33 workers (28.2%), respectively. Mean LDS values for mild, moderate, and severe categories were 3.3 (n = 8), 9.9 (n = 76), and 19.9 (n = 33), respectively. These subjects had been working in sandstone mines and stone quarries. The radiographs of 32 healthy controls were within normal limits. Mean serum CC16 value of control population was 16.3 ± 3.8 ng/mL. It was observed that serum CC16 values were significantly decreased in relation to increasing lung damage (ie, mild, moderate, and high) among the study subjects. The mean serum CC16 value in mild lung damage category was 8.4 (SD ± 0.87) ng/mL as compared to 4.0 (SD ± 2.1) ng/mL in moderate and 0.7 (SD ± 0.21) ng/mL in high or severe lung damage categories (Figure 1). It is interesting to note that duration of exposure is inversely associated with the amount of lung damage in this study. This may be attributed to the fact that varying quantity of dust with varying silica content may be inhaled by them over the years (Table 1). Differences of mean of various categories of LDS were found to be statistically significant (moderate vs mild and high vs moderate) as indicated by *P* = <.01 (Table 1). Similarly, differences of mean value of serum CC16 levels in various lung damage categories were found to be statistically significant (moderate vs mild and high vs moderate) indicated by *P* = <.01 (Table 1).

**TABLE 1:** Age, duration of exposure, and lung damage category with score and serum CC16

Parameters Mean ± SD	Healthy (LDS = 0) (n = 32) [Mean ± SD]	Mild lung damage (n = 8) [Mean ± SD]	Moderate lung damage (n = 76) [Mean ± SD]	High lung damage (n = 33) [Mean ± SD]
Age (yr)	37.5 ± 11.41	50.5 ± 13.84	46.2 ± 9.87	47.5 ± 3.91
Duration of exposure (yr)	Nil	28.6 ± 11.26	21.0 ± 9.95	19.8 ± 4.00
Lung damage score (LDS)	Nil	3.3 ± 1.00	9.9 ± 3.72^a^	19.9 ± 1.03^b^
Serum CC16 (ng/mL)	16.3 ± 3.8	8.4 ± 0.87	4.0 ± 2.10^c^	0.7 ± 0.21^d^

^a^ *P* = <.01 for moderate and mild lung damage group.

^b^ *P* = <.01 for high and moderate lung damage group.

^c^ *P* < .01 for serum CC16 value for moderate and mild lung damage group.

^d^ *P* < .01 for serum CC16 value for high and moderate lung damage group.

Abbreviations: LDS, lung damage score; SD, standard deviation.

## 4 DISCUSSION

### 4.1 Serum CC16—a possible biomarker for secondary prevention

The study observed that most silicotic subjects were detected when they had moderate lung damage (65%; n = 76) followed by severe lung damage (28.2%; n = 33). Only 6.8% (n = 8) of the studied workers were detected with mild lung damage (Figure 1). This evidences that most silicotic subjects are detected when lung damage progresses to the extent of moderate to severe stage. The results also indicated an inverse relationship between the extent of lung damage and the serum CC16 value. Differences of mean serum CC16 values from healthy to mild lung damage, mild to moderate damage, and moderate to high damage are statistically significant suggesting serum CC16 might be used as a screening tool for early detection of silicosis cases among silica dust exposed workers. Earlier studies showed that smoking reduced serum CC16 to slight extent, and cessation of smoking elevates serum CC16 level.^12^, ^14^, ^15^ None of the study participants was current smoker in this study. So, adjustment of serum CC16 value related to smoking habit was not required. It was observed that most silicosis patients quit their smoking habits following detection of silicosis on medical advice or when they develop distressing respiratory symptoms such as dry or productive cough with suspicion by the health care workers during their first consultation with them. Serum CC16 levels are inversely proportional to the amount of lung damage among silica dust–exposed workers as observed in this study (Figure 1). So, if screening is performed in selected population with history of silica dust exposure, serum CC16 appears to be useful for early detection of silicosis as evidenced in this study as well as in earlier study. The present study was conducted among the workers with history of occupational exposure to silica dust. The dusty workplace exacerbates symptoms of bronchial asthma, chronic bronchitis, and COPD, and hence the persons suffering from such medical conditions cannot sustain for longer duration in dusty environment and eventually leave the job. It was also observed in our earlier study that serum CC16 value does not vary with age and/or gender. Hence, it could be used as a screening tool for early detection among selected group (having history of silica dust exposure) by periodic assessment. Progression of silicosis depends on silica content of the inhaled dusts, duration, and amount of dust exposure in a day, week, year, etc with or without use of protective devices, etc. This probably explains the fact of more lung damage with lesser duration of work in some workers as observed in this study.

This study also indicates that the lung damage may be categorized to mild, moderate, and severe based on the declining level of serum CC16 value among silicotic subjects; mild lung damage = >6 to 9 ng/mL, moderate lung damage = >3 to 6 ng/mL, and severe lung damage = 3 ng/mL or less. Hence, early silicosis may be considered if serum CC16 value remains between >6 and 9 ng/mL. Since smoking reduces CC16 value by 1 to 2 ng/mL, above range will include both smokers as well as nonsmokers for screening purpose from operational point of view under a public health programme. Some workers might have silica dust exposure history as well as serum CC16 value of just above 9 ng/mL. They may be in their very early pathological process of silicosis. But usually, these subjects are not detectable by chest X‐ray, hence, unsuitable for confirmation. Considering all these, 9 ng/mL may be the cut‐off value for detection of silicosis at an early stage.

In a country like India and similar other countries where burden of silicosis is high, periodic estimation of serum CC16 value using above‐said method among dust exposed workers will be helpful in reducing silicosis burden by its early detection and effective intervention. On the other hand, silicosis is intimately associated with silico‐tuberculosis due to reduced lung immunity.^16^, ^17^, ^18^ High prevalence of tuberculosis among silica dust–exposed workers has been depicted by other research studies.^19^, ^20^ The findings of earlier study suggested that highest levels of CC16 in X‐ray confirmed silicosis patients were around 9 ng/mL. This study has also showed that silicosis with mid lung damage had mean serum CC16 levels of 8.4 ± 0.87 ng/mL. Hence, the study intends to highlight that early silicosis may be suspected when serum CC16 value is 9 ng/mL or slightly lower provided the subjects are nonsmokers or stopped smoking for at least 1 to 2 months before the test date. This is expected to ensure confirmation of early silicosis by chest X‐ray or high‐resolution CT scan, a clinical and legal requirement as per law of the country. So, serum CC16 may be used as a screening tool for early detection of silicosis by periodic screening among workers with history of silica dust exposure. It should not be used for assessing lung health of other category of population without further evaluation.

### 4.2 Policy decision toward control of silicosis

Silicosis is a neglected public health disease with huge burden in India. Most qualified physicians are not in a position to diagnose it properly due to lack of training during their medical practicing tenure or earlier (during medical graduation). Presently, silicosis diagnosis is based on the basis of presence of typical bilateral patchy opacities on chest X‐ray. But, the X‐ray facility is not available in most rural areas in India, and most physicians lack training about detection of silicosis. As a result, silicosis is often wrongly diagnosed and treated as tuberculosis by the treating physicians without much beneficial effects in these underprivileged workers. Considering above along with lack of any effective commitments from the appropriate authorities, necessary initiatives are required for prevention and control of silicosis. India requires to undertake an early policy decision to initiate a national silicosis control programme similar to other national control programmes for diseases of public health interest. It may be noted that India is already committed to elimination of tuberculosis by 2025. But one needs to understand that unless silicosis is controlled, elimination of tuberculosis is not possible as India has a huge burden of silicosis and silicotic subjects are vulnerable to silico‐tuberculosis. One also needs to understand that the vulnerable workers are either under the ministry of labor or mines or industries, whereas the department of health is under the ministry of health and family welfare. As a result, there is no commitment from the department of health towards these vulnerable workers. Hardly there is any coordination among these relevant departments mentioned above. India has more than 500 million workers, and more than 90% of them are in the informal economy sector, who do not have minimum medical and/or other social benefits except having some small daily wage.^21^ Moreover, a sizable number of workers are migrant in nature and are usually associated with most hazardous work activities. Hence, a suitable legislation is necessary in coordination with relevant departments to enable all vulnerable workers, particularly those of informal economy sectors for prevention and control of silicosis, one of the most prevalent occupational diseases in the country. Considering a huge unemployment problem in the country, particularly among unskilled workers, primary prevention of silicosis is often difficult. But secondary prevention of silicosis by annual screening and early detection using some suitable biomarker such as serum CC‐16 and implemented through the country‐wide network of primary health centers may be a suitable alternative. As observed in this study, serum CC‐16 cut‐off value of 9 ng/mL or slightly below may be used for suspecting an early silicosis. Once silicosis is suspected through periodic screening, confirmation may be carried out by chest X‐ray or HRCT scan as per ILO radiography guidelines for detection of silicosis. Since there are no separate occupational health care delivery services for formal or informal economy sector workers, the existing primary health care delivery services may be prepared adequately to accommodate this additional responsibility supported by regional as well as central health authorities. This includes provision of necessary training of the health care workers and necessary logistic supports to be made available in all peripheral health care establishments to improve the national occupational health scenario focusing control of silicosis and silico‐tuberculosis. Facility of early detection of silicosis can be made available at every primary health care facilities of the country. It may be noted that ICMR—National Institute of Occupational Health in collaboration with ICMR—National Institute of Virology has already developed an indigenous and affordable Point of Care (POC) screening kit for detection of serum CC16 even in remote rural areas, which is currently ready for commercialization. The said kit is expected to be available in the market soon for mass use by the peripheral health care workers. Silicosis is a notifiable as well as compensable disease. Once silicosis is diagnosed, a lump sum amount of money is given to the victim as compensation, but there is no system for stopping the victim from further exposure or providing him alternative employment opportunity. This results in continuation of similar work with continued silica dust exposure either in same or in other similar work place. Considering the lack of commitment to workers' health and safety in India, a suitable national silicosis control programme is urgently needed focusing secondary prevention using a suitable biomarker such as serum CC16 to save/prolong the lives of the vulnerable workers. Moreover, this will prevent not only silicosis by stopping/minimising further dust expose but also its associated co‐morbidity, silico‐tuberculosis. Silico‐tuberculosis could detected at early stage by periodic screening of silicotic workers' sputum using CBNAAT/True‐NAAT. We understand that the same holds true for other similar countries with high burden of silicosis such as South Africa, China, Brazil, Thailand, Australia, some European countries, etc. They would also be benefitted initiating similar silicosis intervention programme in their countries using CC16 or similar marker for early detection as suggested by a recent study.^22^ International organizations such as WHO, The Union, etc, should come forward coordinating and guiding member countries and taking this matter forward further in order to achieve the desired goal. The concept of secondary prevention of silicosis using an effective screening tool such as serum CC16 may be validated in above‐said countries to establish its potential benefits both nationally as well as internationally.

Finally, a national silicosis control programme is an immediate need of the country. Institute/s having technical expertise on silicosis and silicos‐tuberculosis such as ICMR—National Institute of Occupational Health may be considered as a nodal agency for supervision and monitoring of national silicosis control activities of the country in coordination with other relevant institutes. Both formal as well as informal economy sector workers need to be screened annually using CC16 as a proxy marker for early detection of silicosis. A suitable legislation is required toward this. All silicotic subjects need to be screened periodically for early detection of silico‐tuberculosis along with undertaking necessary intervention measures for strengthening country's tuberculosis elimination activities. It is also suggested to maintain a proper registry of all detected silicotic subjects, who need to be linked with some unique identifier (such as Aadhar no. in India) for tracking them longitudinally to avoid loss to follow‐up as many of them work as migrant workers. All primary health centers need to be equipped with point of care, user's friendly CC16 kit, and all primary health care workers need to be trained for screening of silica dust–exposed workers using CC16 kit. Community awareness about silicosis and silico‐tuberculosis involving relevant workers and their family members, social activists, union leaders, industry owners, village leaders, etc, is needed. Suitable medical curriculum to train medical and para‐medical workers on basic occupational health and safety is an essential component. All relevant departments such as health, labor, mines, industries, etc, need to work together in a coordinated fashion under leadership of a coordinating committee consisting of representative members of above departments.

### 4.3 Limitation of the study

Following are the limitations of the present study:

Inclusion of a smaller number of mild lung–damaged subjects as compared to moderate and advanced cases. It could be due to the fact that early‐stage silicosis is mostly asymptomatic and ignored, and hence majority are diagnosed when silicosis is in moderate or in advanced stages. Hence, early silicosis with mild lung–damaged subjects were few. Self‐reported behavior of the study subjects including smoking habits. Lack of some other relevant information such as relevant behaviors of the subjects facilitating silica dust exposure, silica content of exposed dusts, work place hygiene, etc. Concurrent exposure to factors like PAH, PM 2.5, etc, may alter the lung damage status to some extent in addition to silica dust, which could not be evaluated in this study.

## 5 CONCLUSION

Silicosis is a neglected occupational disease and associated with high morbidity and premature mortality. The effective initiatives toward its prevention and control in India is practically non‐existent. Considering its huge burden, the country needs an urgent policy decision toward initiating national silicosis control programme for dual benefit of control of both silicosis as well as silico‐tuberculosis. Secondary prevention of silicosis and silico‐tuberculosis by using a suitable and cost‐effective biomarker such as CC16 may be viewed as a new approach of the proposed national silicosis control programme.

## FUNDING

Intramural funding from ICMR—National Institute of Occupational Health.

## CONFLICT OF INTEREST

The provision of financial support does not in any way infer or imply endorsement of the research findings by either agency. The authors declare no conflict of interest relating to the material presented in this article. Its contents, including any opinions and/or conclusions expressed, are solely those of the authors.

## AUTHORS' CONTRIBUTIONS

Conceptualization: Kamalesh Sarkar.

Formal Analysis: Pankaja Raghav, Umesh Chandra Ojha, Bidisa Sarkar.

Funding Acquisition: Kamalesh Sarkar.

Laboratory Work: Avinash Paghdhune.

Supervision of clinical work: Umesh Chandra Ojha.

Writing—Original Draft Preparation: Kamalesh Sarkar.

Writing—Review and Editing: Bidisa Sarkar, Pankaja Raghav, Sarang Dhakrak.

The manuscript was prepared in consultation with all authors, and final draft to be published was approved by all authors.

Manuscript has been read and approved by all the authors, that the requirements for authorship as stated earlier in this document have been met, and that each author believes that the manuscript represents the sincere and honest work.

## TRANSPARENCY STATEMENT

The lead author (Kamalesh Sarkar) affirms that the manuscript is an honest, accurate and transparent account of the study being reported and no important aspects of the study have been omitted. Also declares that any discrepancy from the study as planned (if relevant) have been explained.

## ETHICS STATEMENT

The study was duly reviewed and approved by Institutional Ethics Committee (IEC) of National Institute of Occupational Health.
